# Carotenoids and Periodontal Infection

**DOI:** 10.3390/nu12010269

**Published:** 2020-01-20

**Authors:** Koji Naruishi

**Affiliations:** Department of Periodontology and Endodontology, Tokushima University Graduate School of Biomedical Sciences, 3-18-15 Kuramoto, Tokushima 770-8504, Japan; naruishi@tokushima-u.ac.jp; Tel.: +81-88-633-7344; Fax: +81-88-633-7345

**Keywords:** carotenoids, periodontitis, low-grade infection

## Abstract

Periodontitis is a polymicrobial infectious disease that leads to inflammation of the gingiva, resulting in teeth loss by various causes such as inflammation-mediated bone resorption. Recently, many investigators have reported that the periodontitis resulting from persistent low-grade infection of Gram-negative bacteria such as *Porphyromonas gingivalis* (*Pg*) is associated with increased atherosclerosis, diabetes mellitus, and other systemic diseases through blood stream. On the other hand, carotenoids belong among phytochemicals that are responsible for different colors of the foods. It is important to examine whether carotenoids are effective to the inhibition of periodontal infection/inflammation cascades. This review summarizes the advanced state of knowledge about suppression of periodontal infection by several carotenoids. A series of findings suggest that carotenoids intake may provide novel strategy for periodontitis treatment, although further study will be needed.

## 1. Introduction

Periodontitis is a chronic inflammatory disease affecting the supporting tissues of teeth [1]. The disease is caused by infection of specific microorganisms such as *Porphyromonas gingivalis* (*Pg*), which causes progressive destruction of the alveolar bone. More than 500 individual species of microbes have been identified in the human mouth [2], and the infection with periodontal bacteria causes humoral immunological/inflammatory responses [3]. Furthermore, periodontal inflammation induced by the bacterial infection exacerbates alveolar bone resorption. Since bone homeostasis is regulated by a balance of osteoblastic bone formation and osteoclastic bone resorption, the functional imbalance between osteoblasts and osteoclasts leads to the progression of periodontitis [1].

Recently, many reports showed that persistent low-grade infection of *Pg* in periodontitis lesions is associated with increased atherosclerosis, diabetes mellitus, and other systemic diseases disseminated through the blood stream [4,5]. Therefore, control of periodontal infection is not only important for oral health, it may also contribute to improve overall health. Clinically, the infection control by the adjunctive use of systemic or local antibiotics provide additional benefits in the treatment of periodontitis. However, dentists must consider the increase of risk of complications associated with antibiotics abuse, particularly those related to antimicrobial resistance. The use of natural antioxidant substances on treatment of this high prevalence disease instead of antibiotics may be effective.

Carotenoids belong among the phytochemicals that are responsible for different colors of foods [6]. It is well known that these natural diet components are widely found in many fruits and vegetables, and exert a rich variety of physiological benefits and are beneficial for human health. This review summarizes the advanced knowledges about suppression of periodontal infection by carotenoids and the possibility of clinical use will be discussed.

## 2. Pathogenesis of Periodontitis

Periodontitis is a bacterial infectious disease, and inflammation cascades in the periodontal lesions regulate the disease pathogenesis [7,8]. Roles of inflammatory cytokines such as interleukin (IL)-1β and IL-6 in periodontitis have been explored by targeting fibroblasts, epithelial cells and macrophages [9,10]. Both IL-1β and IL-6 cause tissue destruction by inducing the production of matrix-metalloproteinase-1 (MMP-1) in inflamed periodontal tissues [11]. MMP-1 is released into the inflamed tissues, and destroys the connective tissues by degrading collagen directly or by activating the fibrinolytic protease cascades because type I collagen is accumulated mainly in periodontal tissues [11,12]. Imbalance of MMPs and the inhibitors such as tissue inhibitors of MMPs (TIMPs) induces pathological degradation of the collagens fiber in inflamed periodontal tissues [13].

**Figure 1:**
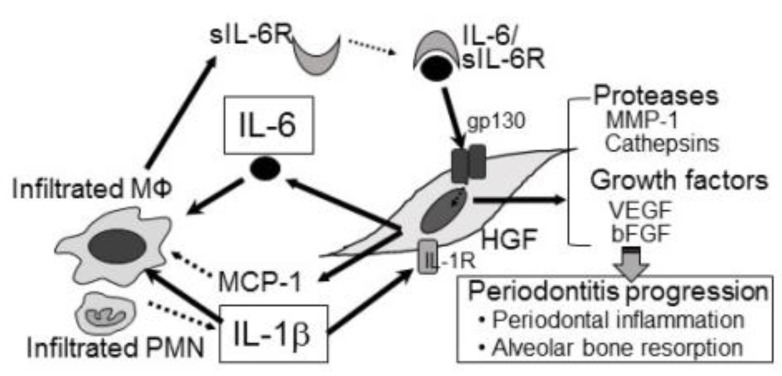
Crosstalk of HGFs and inflammatory cells: Potential biological mechanisms of periodontitis. In inflamed periodontal tissues, IL-1 induces sIL-6R production in infiltrated inflammatory cells such as M. Furthermore, IL-1 induces production of IL-6 in HGFs. Finally, IL-6/sIL-6R complexes induce MMP-1, cathepsins, bFGF and VEGF production in HGFs, resulting in progression of periodontitis.

Human gingival fibroblast (HGF) is an important abundant cell in periodontal tissues [14]. Although the remodeling of periodontal connective tissues is main role of HGFs, HGFs also regulates the inflammation cascades in periodontitis lesions [15,16]. In addition, Holden et al., reported that resident macrophages produce tumor necrosis factor-α (TNF-α) and IL-10 in response to the subgingival microorganisms such as fimbria and lipopolysaccharide (LPS) [17]. Thus, macrophages have been involved in the inflammatory responses of periodontitis [18]. Cytokine balance regulated by a crosstalk between tissue cells and immune cells plays important roles in the stability and progression of the disease (Figure 1).

### 2.1. Proteases and Periodontitis

Many proteases induce the degradation of extracellular matrix in periodontitis lesions, and the proteases contain MMPs and cysteine proteases, i.e., cathepsin B and L [19]. As described above, MMP-1 is released into inflamed periodontal tissues, and may be involved in the destruction of collagen fibers. Sawada et al., reported that MMP-1 production increased significantly in HGFs treated with IL-1β and IL-6/sIL-6R [11]. On the other hand, cathepsin B and L are involved in both intracellular proteolysis and extracellular matrix degradation so that the proteases induce gingival tissue destruction [20]. In addition, although cathepsin B degrades collagen fibers directly, the cathepsin B also contributes to collagen degradation indirectly through activation of MMP-1 [19]. Previously, it has been shown that levels of cathepsin B and L increase in the gingival crevicular fluids (GCFs) of patients with periodontitis [21]. We reported previously that IL-6/sIL-6R induced significantly cathepsin B and cathepsin L secretion in HGFs [22]. Therefore, the proteases such as MMP-1, cathepsin B and L released from HGFs treated with both IL-1β and IL-6/sIL-6R might act cooperatively in degradation of periodontal tissues. In general, although MMPs work at neutral pH, the local area in inflamed lesion has an acidic pH at attachment sites of macrophages and osteoclasts [23]. Since chronic periodontitis is one of local inflammatory diseases with bone resorption, local acidic conditions may be emphasized the action of cathepsins rather than MMPs in periodontitis lesions.

### 2.2. Chemokines and Periodontitis

Chemokines such as IL-8 and MCP-1 are chemoattractant factors for polymorphonuclear leucocytes (PMNLs), and have an important role in the pathogenesis of periodontitis [24,25]. PMNLs play a role in the first defense against microbial invasion in the body. It is well known that the PMNLs such as neutrophils help in controlling the microbial invasion by several intracellular and extracellular oxidative killing mechanisms, i.e., formation of reactive oxygen species (ROS) [25]. It has been reported that GCF levels of both IL-8 and MCP-1 is significantly higher in patients with periodontitis than in periodontally healthy controls. We showed previously that IL-1β and IL-6/sIL-6R induced MCP-1 production from HGFs synergistically [11]. Furthermore, IL-1β induced IL-8 production in HGFs, although IL-6/sIL-6R did not induce the IL-8 production. Although specific roles of IL-8 and MCP-1 are still unknown pathologically, the onset of periodontitis may be regulated directly by these chemokines.

### 2.3. Growth Factors and Periodontitis

Angiogenesis, an important process of new blood vessel formation, induces the progression of periodontitis [26]. Although several angiogenic factors have been known, VEGF is the most powerful inducer of angiogenesis [27]. Our previous reports have shown that IL-1β and IL-6/sIL-6R induced the production of VEGF from HGFs [11,16]. Increased VEGF from HGFs would be a key inducer in the severity of periodontitis. On the other hand, it has been reported that cathepsin B in vasculature increased dramatically during the degradation of vascular basement membrane in tumor-mediated angiogenesis [28]. Furthermore, Yanamandra et al., reported that knockdown of cathepsin B gene in glioma cells suppressed significantly the tumor-mediated angiogenesis by inhibition of VEGF production [29]. Therefore, VEGF and cathepsin B secreted by HGFs should induce the angiogenic cascades in periodontitis lesions cooperatively by affecting angiogenesis-associated cells such as endothelial cells. In addition, basic FGF (bFGF) is also a strong angiogenic inducer, and can induce the migration or growth of endothelial cells, resulting in formation of the capillary tubes [30]. We reported previously that IL-1β and IL-6/sIL-6R enhanced bFGF secretion synergistically in HGFs [11]. Interestingly, the synergistic effects depend on IL-1β-induced gp130 expression in cell membrane of HGFs. Gp130 regulates the signaling pathway of many cytokines such as IL-6, IL-11, ciliary neurotrophic factor, leukemia inhibitory factor, oncostatin M [31]. These cytokines are involved in many biological responses including inflammation, immune responses and cell growth [32]. Therefore, IL-1β-mediated increase of gp130 in HGFs will be attractive target for regulation of inflammation cascades in periodontitis lesions.

## 3. Carotenoids and Periodontitis

Carotenoids, as dietary antioxidants, have inhibitory effects on the progression of inflammatory diseases. Carotenoids neutralize the ROS activation that can induce oxidative stress, which results in excessive tissue damages. Carotenoids can protect the damages of tissue cells from unwanted several diseases induced by inflammation such as periodontitis or aging.

The main question of this review was: is there a relationship between carotenoids and periodontitis? I used the MEDLINE-Pubmed databases for search of this review, and the search strategy included the following terms: “carotenoids” (MeSH Terms) OR “carotenoids” (All Fields) AND “periodontitis” (MeSH Terms) OR “periodontitis” (All Fields). The literature was searched up to December 2019. As a result of the search, 42 titles/abstracts were retrieved, and 41 of the selected articles were written in English. Although carotenoids are known as playing an important role in the prevention of inflammatory diseases, the clinical efficacy for periodontitis may be only beginning to be investigated now (Table 1).

### 3.1. β-Carotene

β-Carotene, one of the main carotenoids, is a vitamin A precursor that has anti-oxidant or anti-cancer effects. In patients with diabetes, higher blood β-carotene levels, associated with green or yellow vegetables intake, confer good effects against insulin resistance [45]. Amengual et al., also reported that β-carotene reduced the adiposity of body, downsize of adipocytes and blood leptin levels using in vivo experiment [46]. Importantly, Ebersole et al., have shown that lower β-carotene levels in blood were found in moderate/severe periodontitis patients [40].

In general, cellular responses to high glucose in inflamed periodontal tissues are thought to induce the development of diabetic complications [5]. Increased low-grade persistent inflammation mediated by invasion of periodontal bacteria into blood stream is a key factor in the development of diabetic complications. Importantly, we showed that β-carotene suppressed significantly the *Pg* LPS-induced TNF, IL-6 and MCP-1 production in THP-1 monocytes cultured with high glucose conditions via NF-kB signaling without cell damages [33]. Therefore, β-carotene may be useful to interfere with diabetic complications by targeting intracellular NF-kB. The patients with diabetes might be able to prevent or delay the periodontitis-mediated development of several complications by the appropriate dietary treatment using β-carotene. In addition, it has been reported that dietary intakes of β-carotene were associated with reduced periodontal pocket depth after periodontal treatment in nonsmokers, but not smokers, with chronic periodontitis [41]. Since β-carotene intake may be effective to the impairment of inflammation responses in both local and systemic area, the importance of dietary strategies to optimize healing after periodontal therapy should be discussed in the future.

### 3.2. β-Cryptoxanthin

β-Cryptoxanthin, one of the main carotenoids, is found abundantly in fruit and vegetables such as papaya, paprika, and carrot [47]. β-Cryptoxanthin also exerts various biological activities such as antioxidant functions. Ebersole et al., have reported that lower β-cryptoxanthin levels in blood were found in moderate/severe periodontitis patients [40]. Interestingly, Hirata et al., reported that β-cryptoxanthin suppressed LPS-induced osteoclasts differentiation via PGE_2_ inhibition in HGFs and restored the alveolar bone loss in an experimental in vivo periodontitis models [47]. In addition, Matsumoto et al., reported that β-cryptoxanthin suppressed the osteoclast formation mediated by LPS in co-cultures of osteoblasts and bone marrow cells [34]. They have also mentioned that β-cryptoxanthin inhibited the levels of LPS-induced alveolar bone resorption significantly in experimental in vivo periodontitis models. Furthermore, Nishigaki et al., reported that β-cryptoxanthin reduced significantly *Pg*-induced production of IL-6 and IL-8 in human periodontal ligament cells [35]. A series of these reports support the notion that β-cryptoxanthin is an effective carotenoid for the treatment of periodontitis.

### 3.3. Astaxanthin

Astaxanthin has a variety of biological activities such as antioxidant effects or inhibitory effects against asthma or neuro-inflammation [48]. Recently, Hwang demonstrated that anti-osteoporotic effects of astaxanthin on the bone mass of ovariectomized (OVX) mice [48]. Interestingly, administration of astaxanthin suppressed significantly the increase of serum calcium levels, alkaline phosphatase and tartrate-resistant acid phosphatase (TRAP) activity in the OVX mice. Importantly, the bone mineral density of trabecular bone in both tibia and femur were recovered by astaxanthin. Furthermore, astaxanthin suppressed the osteoclast formation through the regulation of nuclear factor of activated T cells (NFAT) c1. Balci et al., reported previously that astaxanthin administration reduced alveolar bone resorption by enhancing the osteoblastic activity, and decreased osteoclastic activity in experimental ligature-induced periodontitis models [37]. They have also shown that astaxanthin decreased TRAP-positive osteoclasts and increased number of osteoblasts. Since periodontitis occurs alveolar bone loss, astaxanthin may be useful in periodontitis patients with postmenopausal osteoporosis. Taken together, astaxanthin administration may be effective as a new treatment for periodontitis.

### 3.4. Fucoxanthin

Fucoxanthin inhibits RANKL-mediated osteoclastogenesis [38]. Kose et al., reported previously that fucoxanthin resulted in a slight decrease in blood TNF, IL-1β and IL-6 levels using experimental in vivo periodontitis models [38]. Although fucoxanthin is effective to the suppression of periodontitis-mediated systemic responses, they also mentioned that no significant effects were observed in alveolar bone loss levels of rats with or without fucoxanthin treatment. This study showed that fucoxanthin may provide a limited reduction in alveolar bone resorption in periodontitis. Therefore, fucoxanthin may have a limited inhibitory effect in the bone loss seen in periodontitis.

### 3.5. All-trans Retinoic Acid

All-*trans* retinoic acid is made from vitamin A in the human body [49]. In addition, all-trans retinoic acid regulates the relationship between T-helper 17 cells (Th17) and regulatory Tcells (Treg), resulting in the prevention and treatment of several types of cancer [49]. Furthermore, imbalance of Th17 and Treg plays an important role in the pathophysiology of periodontitis, although the precise mechanisms are still unknown. Importantly, Wang et al., reported that all-*trans* retinoic acid suppressed alveolar bone resorption throughout the inhibition of inflammatory cell infiltration into periodontal tissues in *Pg* LPS-induced experimental periodontitis models [39]. Furthermore, they have also shown that all-*trans* retinoic acid reduced IL-17 levels in the *Pg* LPS-infected periodontal tissues [39]. Progression of periodontitis may be suppressed by all-*trans* retinoic acid-mediated Th17/Treg imbalance induced by both activation of Treg cells and inhibiting Th17 cells. A series of results indicate that the all-*trans* retinoic acid has an important role in immune responses, leading to the prevention of periodontitis.

### 3.6. Lycopene

Lycopene is one of antioxidant carotenoids contained in tomato products mainly [50]. Arora et al., reported that systemic lycopene administration suppressed salivary IL-1 levels, resulting in inhibition of periodontal inflammation [42]. Belludi et al., have also reported that lycopene is an effective carotenoid in patients with periodontitis clinically [43]. Although number of subjects in the clinical study was very limited, significant improvement of periodontal healing was observed after oral prophylaxis in Lycopene-treated group compared with control group. Furthermore, Chandra et al., examined whether there were significant differences between smokers and nonsmokers in the clinical parameters such as periodontal probing depth after lycopene administration [44]. Interestingly, lycopene-treated sites in periodontitis lesions showed significant reductions in the probing depths in both smokers and nonsmokers.

### 3.7. Luteolin

Luteolin, extensively present in many plant species, is one of natural antioxidants [51]. Luteolin has anti-inflammatory, anti-allergic and anti-oxidant effects, and the biological effects could be functionally related to each other. Ohyama et al., reported that interferon gamma induces the histocompatibility leukocyte antigen-DR (HLA-DR) expression on cell membrane of HGFs [52]. HLA-DR cannot present antigens to induce T-cell proliferation in HGFs, but acts as a receptor that activates focal adhesion kinase (FAK) signaling into HGFs, resulting in the secretion of several cytokines [52]. Previously, Yoshizawa et al., showed that luteolin suppressed HLA—DR—Induced IL-6 and MCP-1 production in HGFs via FAK inhibition [36]. Although luteolin is an attractive carotenoid for periodontitis treatment, further investigation will be required to show the efficacy of luteolin on periodontitis.

## 4. Conclusions

Carotenoids are involved in the inhibition of reactive oxygen species. A series of findings suggest that carotenoid intake may provide a novel strategy for periodontitis treatment. However, there are no evident target molecules for carotenoids, although almost carotenoids probably have an anti-oxidant activity. Therefore, effective and safe methods of carotenoid use in the clinical therapy of periodontitis should be considered, after further studies to be performed in the future. In addition, combinations of carotenoids might be more effective than single compounds in preventing oxidative tissue damage, and the synergistic effects should be considered more in depth.

## Figures and Tables

**Table 1:** Summary of the studies: inhibitory effects of carotenoids on progression of periodontitis.

Study Designs	Carotenoids	Target	Inhibitory Effects	Ref
In vitro	β-Carotene	THP-1 monocytes	Inhibition of *Pg* LPS-induced TNF, IL-6 and MCP-1 production	[33]
	β-Cryptoxanthin	Human gingival fibroblasts	LPS-induced osteoclasts differentiation via inhibition of PGE_2_ production	[33]
		Co-cultures of bone marrow cells and osteoblasts	Inhibition of LPS-induced osteoclast formation	[34]
		Human periodontal ligament cells	Inhibition of *Pg*-induced IL-6 and IL-8 production	[35]
	Luteolin	Human gingival fibroblasts	Inhibition of HLA-DR-induced IL-6 and MCP-1 production	[36]
In vivo	β-Cryptoxanthin	Experimental periodontitis	Inhibition of alveolar bone loss	[33]
			Inhibition of alveolar bone loss	[34]
	Astaxanthin		Inhibition of osteoclastic activity	[37]
	Fucoxanthin		Decrease of blood TNF, IL-1β and IL-6 levels	[38]
			No effects of alveolar bone loss	[38]
	All-trans retinoic acid		Decrease of blood IL-17 levels	[39]
			Inhibition of alveolar bone loss	[39]
Clinical study	β-Carotene	NHANES (1999–2004), total *N* = 15,844	Lower blood levels	[40]
		Periodontitis patients, *N* = 86	Adjunctive effects of periodontal therapy	[41]
	β-Cryptoxanthin	NHANES (1999–2004), total *N* = 15,844	Lower blood levels	[40]
	Lycopene	Periodontitis patients, *N* = 42	Inhibition of salivary IL-1 levels	[42]
		Periodontitis patients, *N* = 20	Improvement of periodontal healing	[43]
		Periodontitis patients, *N* = 100	Decrease of periodontal probing depth	[44]

NHANES, The National Health and Nutrition Examination Survey.

## References

[1] Darveau R.P., Tanner A., Page R.C. (2004). The microbial challenge in periodontitis. Periodontol. 2000.

[2] Paster B.J., Olsen I., Aas J.A., Dewhirst F.E. (2006). The breadth of bacterial diversity in the human periodontal pocket and other oral sites. Periodontol. 2000.

[3] Aukhil I., Lopatin D.E., Syed S.A., Morrison E.C., Kowalski C.J. (1988). The effects of periodontal therapy on serum antibody (IgG) levels to plaque microorganisms. J. Clin. Periodontol..

[4] Zelkha S.A., Freilich R.W., Amar S. (2010). Periodontal innate immune mechanisms relevant to atherosclerosis and obesity. Periodontol. 2000.

[5] Moutsopoulos N.M., Madianos P.N. (2006). Low-grade inflammation in chronic infectious diseases: Paradigm of periodontal infections. Ann. N. Y. Acad. Sci..

[6] Rao A.V., Rao L.G. (2007). Carotenoids and human health. Pharmacol. Res..

[7] Birkedal-Hansen H. (1993). Role of matrix metalloproteinases in human periodontal diseases. J. Periodontol..

[8] Hassell T.M. (1993). Tissues and cells of the periodontium. Periodontol. 2000.

[9] Okada H., Murakami S. (1998). Cytokine expression in periodontal health and disease. Crit. Rev. Oral Biol. Med..

[10] Takashiba S., Naruishi K., Murayama Y. (2003). Perspective of cytokine regulation for periodontal treatment: Fibroblast biology. J. Periodontol..

[11] Sawada S., Chosa N., Ishisaki A., Naruishi K. (2013). Enhancement of gingival inflammation induced by synergism of IL-1β and IL-6. Biomed. Res..

[12] Kida Y., Kobayashi M., Suzuki T., Takeshita A., Okamatsu Y., Hanazawa S., Yasui T., Hasegawa K. (2005). Interleukin-1 stimulates cytokines, prostaglandin E_2_ and matrix metalloproteinase-1 production via activation of MAPK/AP-1 and NF-kappaB in human gingival fibroblasts. Cytokine.

[13] Emingil G., Han B., Gürkan A., Berdeli A., Tervahartiala T., Salo T., Pussinen P.J., Köse T., Atilla G., Sorsa T. (2014). Matrix metalloproteinase (MMP)-8 and tissue inhibitor of MMP-1 (TIMP-1) gene polymorphisms in generalized aggressive periodontitis: Gingival crevicular fluid MMP-8 and TIMP-1 levels and outcome of periodontal therapy. J. Periodontol..

[14] Naruishi K., Takashiba S., Chou H.H., Arai H., Nishimura F., Murayama Y. (1999). Role of soluble interleukin-6 receptor in inflamed gingiva for binding of interleukin-6 to gingival fibroblasts. J. Periodontal. Res..

[15] Naruishi K., Takashiba S., Nishimura F., Chou H.H., Arai H., Yamada H., Murayama Y. (2001). Impairment of gingival fibroblast adherence by IL-6/sIL-6R. J. Dent. Res..

[16] Naruishi K., Nishimura F., Yamada-Naruishi H., Omori K., Yamaguchi M., Takashiba S. (2003). C-jun N-terminal kinase (JNK) inhibitor, SP600125, blocks interleukin (IL)-6-induced vascular endothelial growth factor (VEGF) production: Cyclosporine A partially mimics this inhibitory effect. Transplantation.

[17] Holden J.A., Attard T.J., Laughton K.M., Mansell A., O’Brien-Simpson N.M., Reynolds E.C. (2014). *Porphyromonas gingivalis* lipopolysaccharide weakly activates M1 and M2 polarized mouse macrophages but induces inflammatory cytokines. Infect. Immun..

[18] Sima C., Glogauerm M. (2013). Macrophage subsets and osteoimmunology: Tuning of the immunological recognition and effector systems that maintain alveolar bone. Periodontol. 2000.

[19] Reynolds J.J. (1996). Collagenases and tissue inhibitors of metalloproteinases: A functional balance in tissue degradation. Oral Dis..

[20] Cox S.W., Rodriguez-Gonzalez E.M., Booth V., Eley B.M. (2006). Secretory leukocyte protease inhibitor and its potential interactions with elastase and cathepsin B in gingival crevicular fluid and saliva from patients with chronic periodontitis. J. Periodontal. Res..

[21] Trabandt A., Müller-Ladner U., Kriegsmann J., Gay R.E., Gay S. (1995). Expression of proteolytic cathepsins B, D, and L in periodontal gingival fibroblasts and tissues. Lab. Investig..

[22] Yamaguchi T., Naruishi K., Arai H., Nishimura F., Takashiba S. (2008). IL-6/sIL-6R enhances cathepsin B and L production via caveolin-1-mediated JNK-AP-1 pathway in human gingival fibroblasts. J. Cell Physiol..

[23] Hashimoto N., Kawabe T., Hara T., Imaizumi K., Wakayama H., Saito H., Shimokata K., Hasegawa Y. (2001). Effect of erythromycin on matrix metalloproteinase-9 and cell migration. J. Lab. Clin. Med..

[24] Gamonal J., Acevedo A., Bascones A., Jorge O., Silva A. (2000). Levels of interleukin-1beta, -8, and -10 and RANTES in gingival crevicular fluid and cell populations in adult periodontitis patients and the effect of periodontal treatment. J. Periodontol..

[25] Taylor J.J. (2010). Cytokine regulation of immune responses to *Porphyromonas gingivalis*. Periodontol. 2000.

[26] Rosenkilde M.M., Schwartz T.W. (2001). The chemokine system—A major regulator of angiogenesis in health and disease. APMIS.

[27] Ferrara N. (2000). Vascular endothelial growth factor and the regulation of angiogenesis. Recent Prog. Horm. Res..

[28] Nalla A.K., Gorantla B., Gondi C.S., Lakka S.S., Rao J.S. (2010). Targeting MMP-9, uPAR, and cathepsin B inhibits invasion, migration and activates apoptosis in prostate cancer cells. Cancer Gene Ther..

[29] Yanamandra N., Gumidyala K.V., Waldron K.G., Gujrati M., Olivero W.C., Dinh D.H., Rao J.S. (2004). Blockade of cathepsin B expression in human glioblastoma cells is associated with suppression of angiogenesis. Oncogene.

[30] Yuan K., Jin Y.T., Lin M.T. (2000). The detection and comparison of angiogenesis-associated factors in pyogenic granuloma by immunohistochemistry. J. Periodontol..

[31] Demyanets S., Huber K., Wojta J. (2012). Vascular effects of glycoprotein130 ligands—Part I: Pathophysiological role. Vasc. Pharm..

[32] Hirano T., Ishihara K., Hibi M. (2000). Roles of STAT3 in mediating the cell growth, differentiation and survival signals relayed through the IL-6 family of cytokine receptors. Oncogene.

[33] Kajiura Y., Nishikawa Y., Lew J.H., Kido J.I., Nagata T., Naruishi K. (2018). β-carotene suppresses *Porphyromonas gingivalis* lipopolysaccharide-mediated cytokine production in THP-1 monocytes cultured with high glucose condition. Cell Biol. Int..

[34] Matsumoto C., Ashida N., Yokoyama S., Tominari T., Hirata M., Ogawa K., Ugiura M.S., Yano M., Inada M., Miyaura C. (2013). The protective effects of β-cryptoxanthin on inflammatory bone resorption in a mouse experimental model of periodontitis. Biosci. Biotechnol. Biochem..

[35] Nishigaki M., Yamamoto T., Ichioka H., Honjo K., Yamamoto K., Oseko F., Kita M., Mazda O., Kanamura N. (2013). β-cryptoxanthin regulates bone resorption related-cytokine production in human periodontal ligament cells. Arch. Oral Biol..

[36] Yoshizawa S., Meguro M., Ohyama H., Takeuchi-Hatanaka K., Matsushita S., Takashiba S., Nishimura F. (2007). Focal adhesion kinase mediates human leukocyte histocompatibility antigen class II-induced signaling in gingival fibroblasts. J. Periodontal. Res..

[37] Balci Y.H., Lektemur A.A., Gevrek F., Toker H. (2018). Investigation of the effect of astaxanthin on alveolar bone loss in experimental periodontitis. J. Periodontal. Res..

[38] Kose O., Arabaci T., Yemenoglu H., Kara A., Ozkanlar S., Kayis S., Duymus Z.Y. (2016). Influences of Fucoxanthin on alveolar bone resorption in induced periodontitis in rat molars. Mar. Drugs..

[39] Wang L., Wang J., Jin Y., Gao H., Lin X. (2014). Oral administration of all-trans retinoic acid suppresses experimental periodontitis by modulating the Th17/Treg imbalance. J. Periodontol..

[40] Ebersole J.L., Lambert J., Bush H., Huja P.E., Basu A. (2018). Serum nutrient levels and aging effects on periodontitis. Nutrients.

[41] Dodington D.W., Fritz P.C., Sullivan P.J., Ward W.E. (2015). Higher intakes of fruits and vegetables, β-carotene, vitamin C, α-tocopherol, EPA, and DHA are positively associated with periodontal healing after nonsurgical periodontal therapy in nonsmokers but not in smokers. J. Nutr..

[42] Arora N., Avula H., Avula J.K. (2013). The adjunctive use of systemic antioxidant therapy (lycopene) in nonsurgical treatment of chronic periodontitis: A short-term evaluation. Quintessence Int..

[43] Belludi S.A., Verma S., Banthia R., Bhusari P., Parwani S., Kedia S., Saiprasad S.V. (2013). Effect of lycopene in the treatment of periodontal disease: A clinical study. J. Contemp. Dent. Pract..

[44] Chandra R.V., Sandhya Y.P., Nagarajan S., Reddy B.H., Naveen A., Murthy K.R. (2012). Efficacy of lycopene as a locally delivered gel in the treatment of chronic periodontitis: Smokers vs nonsmokers. Quintessence Int..

[45] Higuchi K., Saito I., Maruyama K., Eguchi E., Mori H., Tanno S., Sakurai S., Kishida T., Nishida W., Osawa H. (2015). Associations of serum β-carotene and retinol concentrations with insulin resistance: The Toon Health Study. Nutrition.

[46] Amengual J., Gouranton E., van Helden Y.G., Hessel S., Ribot J., Kramer E., Kiec-Wilk B., Razny U., Lietz G., Wyss A. (2011). Beta-carotene reduces body adiposity of mice via BCMO1. PLoS ONE.

[47] Hirata N., Ichimaru R., Tominari T., Matsumoto C., Watanabe K., Taniguchi K., Hiratam M., Ma S., Suzuki K., Grundler F.M.W. (2019). Beta-Cryptoxanthin inhibits lipopolysaccharide-induced osteoclast differentiation and bone resorption via the suppression of inhibitor of NF-κB kinase activity. Nutrients.

[48] Hwang Y.H., Kim K.J., Kim S.J., Mun S.K., Hong S.G., Son Y.J., Yee S.T. (2018). Suppression effect of Astaxanthin on osteoclast formation in vitro and bone loss in vivo. Int. J. Mol. Sci..

[49] Li D., Wang P., Wang P., Hu X., Chen F. (2019). Targeting the gut microbiota by dietary nutrients: A new avenue for human health. Crit. Rev. Food Sci. Nutr..

[50] Salehi B., Sharifi-Rad R., Sharopov F., Namiesnik J., Roointan A., Kamle M., Kumar P., Martins N., Sharifi-Rad J. (2019). Beneficial effects and potential risks of tomato consumption for human health: An overview. Nutrition.

[51] Imran M., Rauf A., Abu-Izneid T., Nadeemd M., Shariati M.A., Khan I.A., Imran A., ErdoganOrhan I., Rizwan M., Atif M. (2019). Luteolin, a flavonoid, as an anticancer agent: A review. Biomed. Pharmacother..

[52] Ohyama H., Nishimura F., Meguro M., Takashiba S., Murayama Y., Matsushita S. (2002). Counter-antigen presentation: Fibroblasts produce cytokines by signalling through HLA class II molecules without inducing T-cell proliferation. Cytokine.

